# Benchmarking the stability of human detergent-solubilised voltage-gated sodium channels for structural studies using eel as a reference

**DOI:** 10.1016/j.bbamem.2015.03.021

**Published:** 2015-07

**Authors:** Daria Slowik, Richard Henderson

**Affiliations:** Medical Research Council, Laboratory of Molecular Biology, Francis Crick Avenue, Cambridge CB2 0QH, United Kingdom

**Keywords:** Human voltage-gated sodium channel, Rat VGSC, *Electrophorus electricus*, Membrane protein stability, Structural biology, Radioligand-binding assay

## Abstract

With the ultimate goal of detailed structural analysis of mammalian and particularly human voltage-gated sodium channels (VGSCs), we have investigated the relative stability of human and rat VGSCs and compared them with electric eel VGSC. We found that NaV1.3 from rat was the most stable after detergent solubilisation. The order of stability was rNaV1.3 > hNaV1.2 > hNaV1.1 > hNaV1.6 > hNaV1.3 > hNaV1.4. However, a comparison with the VGSC from *Electrophorus electricus*, which is most similar to NaV1.4, shows that the eel VGSC is considerably more stable in detergent than the human VGSCs examined. We conclude that current methods of structural analysis, such as single particle electron cryomicroscopy (cryoEM), may be most usefully targeted to eel VGSC or rNaV1.3, but that structural analysis on the full spectrum of VGSCs, by methods that require greater stability such as crystallisation and X-ray crystallography, will require further stabilisation of the channel.

## Highlights

• We analysed thermostability of solubilised voltage-gated sodium channels (VGSCs).
• VGSCs are the most thermostable in CHAPS or LMNG within detergents tested.
• VGSCs from electric eel and ratNaV1.3 are more stable than human VGSCs tested.
• NaV1.2 was found to be the most thermostable within human VGSCs tested.
• VGSCs can be stabilised by tetrodotoxin and by sea anemone toxin.

## Graphical abstract

**Figure:**
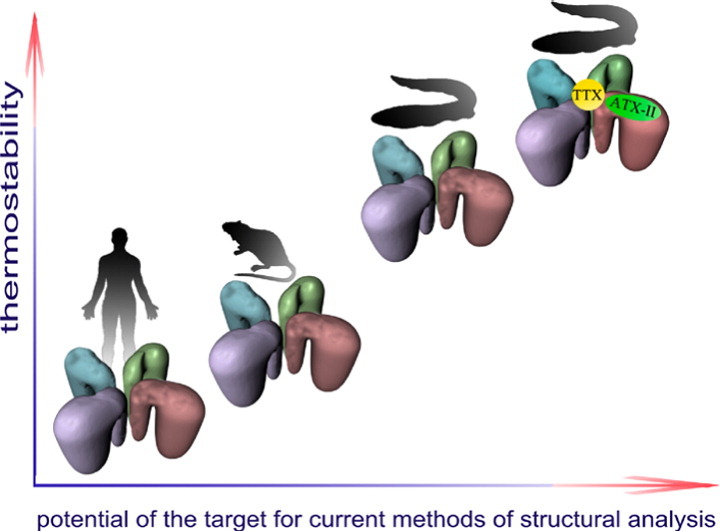

## 1 Introduction

Voltage-gated sodium channels (VGSCs) are dynamic membrane proteins [1] that are selectively permeable to Na^+^ ions when the channels are open. They are crucial for the generation and propagation of action potentials in electrically excitable tissues like muscle, heart, and nerve. Mutations of VGSCs cause multiple inherited diseases known as channelopathies [2,3], including periodic paralysis, cardiac arrhythmia, epilepsy, and neuropathic pain [4–6]. They are key medical targets for local anaesthetic or pain-alleviating drugs as well as being the sites of action of dangerous neurotoxins. Although the kinetics of VGSCs have been extensively characterised *via* electrophysiology [7], and there have been a number of early studies to characterise their secondary [8–11] and tertiary [12] structure, and to model aspects of their detailed three-dimensional (3D) structure [9,13,14], high-resolution structural information remains scanty. High-resolution structures will be essential to extend our understanding of the molecular mechanism of gating (activation and inactivation driven by a drop in the membrane potential) from bacterial to the full range of eukaryotic VGSCs, and for development of therapeutically useful drugs. High resolution structural studies would enable the development of a completely new class of drugs for alleviation of neuropathic pain or anaesthesia.

Because of their key role in human physiology, the VGSC family represents a very important area of research for structural biology. There are, however, many difficulties and challenges associated with structure analysis of mammalian VGSCs *in vitro*. We cannot emphasize strongly enough how difficult it has been even to reach the stage where structural analysis can be attempted on mammalian VGSC. Expression of quantities sufficient for structural work is one problem, but stability is probably the single most important barrier to progress. A membrane protein or membrane protein complex must be sufficiently stable for extraction from its native lipid membrane environment into, at least initially, a detergent-solubilised form. Purification often by a factor of several thousands is then required when expression levels or natural abundance levels are very low, as with VGSCs. Because their normal function in depolarizing excitable membranes requires only a few channels per square micron of membrane, VGSCs have been under strong evolutionary pressure to maintain expression levels at an appropriate and stably regulated low level. Studies have shown [15] that the VGSC α-subunits remain as a metabolically stable intracellular pool of intracellular subunits, whose cell surface expression in active form is regulated through co-expression and combination with β-subunits, especially β_2_. Even while using the mildest detergents and with the addition of lipid or cholesterol-based stabilizing additives, there are classes of eukaryotic membrane protein that are intrinsically unstable once extracted from their natural membrane environment. One of the most successful strategies has been to combine systematically a number of stabilizing mutations to obtain a modified protein that has been thermostabilised, while still retaining its native structure or at least a well-defined conformation that represents one of its natural conformations. This strategy has been termed conformational thermostabilisation [16,17] and has been outstandingly successful when applied to members of the G protein-coupled receptor (GPCR) family [18–21]. However, that approach depends on having a robust assay and expression levels that are sufficient to characterize many hundreds of mutations. We have not yet reached this stage for any human or eukaryotic VGSC.

Even once expression, purification and a degree of stabilization have been obtained, crystallization of the membrane protein is still required before X-ray analysis can be carried. There is a very clear correlation between the stability of a membrane protein in detergent solution and the likelihood of obtaining well-ordered crystals [22]. Alternatively, plunge-freezing is required to allow single particle electron cryomicroscopy [23] to be carried out, and this needs the creation of a thin film with a much larger air–water interface than in bulk solution. The conditions required for crystallization or plunge-freezing often do not coincide with the conditions for greatest stability. For example, addition of exogenous lipids often stabilizes the activity of a membrane protein or channel, but this increases the micelle size and makes crystallization or production of a monodisperse particle, e.g. for electron cryomicroscopy, more difficult. Whatever strategy is being used for stabilization, it is a prerequisite to start off with the most stable representative family member. This is the main purpose of the work we report here.

Nine mammalian isoforms (α-subunits, NaV1.1–NaV1.9) of VGSCs have been identified in the human genome encoded by distinct genes (*SCN1A*-*SCN9A*), but no high-resolution structural analysis has been obtained. Moreover, no expression or purification protocol, which would be suitable for structural studies of human VGSCs has been reported so far, so the possibility of progress on the structure determination of human VGSCs still appears to be some way off. Since VGSCs show strong sequence conservation across species and tissue-specific types [24], much of the early biochemistry on VGSCs, including the determination of the gene sequence [25], has focused on channels from *Electrophorus electricus* electroplax, where these channels constitute approximately 1% of the membrane protein. Channels from *E. electricus* are 59% identical with that of VGSCs from human muscle (hNaV1.4), 51% identical to the human heart channel (hNaV1.5), and 50% identical to the human channels located in the central and peripheral nervous systems (hNaV1.1 and hNaV1.7, respectively). The patterns of hydrophobicity and essential residues are even more closely preserved, increasing the probability that all VGSCs share very similar 3D structures [13,26].

The primary sequence shows that the sodium channel α subunit consists of four evolutionarily homologous internal repeats (domains I–IV), each of which is predicted to consist of six-transmembrane-spanning segments (S1–S6), plus linking regions of differing lengths between the domains and extended aqueous-soluble N- and C-terminal regions. Only a low resolution (19 Å) 3D map of the channel from *E. electricus* determined by single particle cryoEM has been reported [12] and this work was carried out prior to the introduction of more rigorous tests [28,29]. The map appears to show a bell-shaped molecule composed of four domains with pseudo-4-fold symmetry surrounding a central pore. However, with recent progress in validation methods [27,28], and improvements in cryoEM [30], electron detection [31] and computation [32], it should be possible to obtain a more reliable structure. Although the structure would be expected to have many of its core features in common with the known, much simpler structures of bacterial voltage-gated sodium channels [2] or eukaryotic voltage-gated potassium channels [33], the human VGSCs are more than twice the size of these proteins so there is still a great deal of missing information. Human or mammalian VGSCs are also important targets for drug development because of their central role in cardiac function (NaV1.5) and pain transmission (NaV1.7 and NaV1.8). They consist of almost 2000 residues folded into a single extensively glycosylated peptide with a total molecular mass of 260–270 kDa [26,34]. Finally the many toxin binding sites in mammalian VGSCs have no bacterial counterpart.

With the ultimate goal of detailed structural analysis of mammalian and particularly human VGSCs, we have therefore investigated the relative stability of detergent-solubilised human and rat VGSCs and compared them with electric eel VGSC.

## 2 Materials and methods

### 2.1 Materials

Electric eels were obtained from Amazon Fish Direct (UK). Membranes of Chinese hamster ovary (CHO) containing recombinant human VGSCs (hNaV1.1, hNaV1.2, hNav1.3, hNaV1.4 or hNaV1.6) were obtained from ChanTest®, USA), and Human Embryonic Kidney HEK-293 cells stably expressing rat NaV1.3 (rNaV1.3) were kindly provided by Stephen G. Waxman (Yale University, USA). Detergents: 3-[(3-Cholamidopropyl)dimethylammonio]-1-propanesulfonate (CHAPS), *n*-dodecyl-β-D-maltoside (DDM), *n*-decyl-β-D-maltoside (DM), Genapol® C-100 (genapol), and Lubrol-PX (lubrol) were obtained from Sigma-Aldrich Co., and lauryl maltose neopentyl glycol (LMNG) from Anatrace Complete protease inhibitor cocktail tablets (EDTA-free) were obtained from Roche, 3-(N-morpholino)propanesulfonic acid (MOPS) from Fisher Scientific, and 2-({2 [Bis(carboxymethyl)amino]ethyl}(carboxymethyl)amino)acetic acid (Na_2_-EDTA) from VWR International. 11-^3^H saxitoxin (11-^3^H STX, 20 Ci/mmol) was obtained from American Radiolabeled Chemicals, Inc., and tetrodotoxin with citrate (TTX) from Tocris Bioscience or Alomone Labs. Saxitoxin (STX), batrachotoxin (BTX), brevetoxin (PbTx-2), kurtoxin (KTX), veratridine (VTD), tarantula ProTx-II (ProTx-II), and sea anemone toxin (ATX-II) were obtained from Sigma-Aldrich, and huwentoxin-IV (HuwTX) and hainantoxin-III (HaTX) from Alomone Labs Ltd. Quik-sep disposable columns, mini-disposable columns, and Zeba Micro Spin Desalting Columns 7K MWCO for ligand binding assays were from Advanced Laboratory Supplies, BioRad Laboratories Inc., and from Thermo Scientific, respectively. Sephadex G25F, and CM Sepharose were obtained from Amersham Pharmacia, and OptiPhase SuperMix from Perkin Elmer.

### 2.2 Isolation of electric eel membranes

Medium-sized eels (length 0.7–1.5 m) were killed by decapitation after hypothermia. The electroplax organ was dissected, cut into 2-cm slices and stored in liquid nitrogen. Thawed electroplax tissue was homogenised for 30 s with an HR1610/01 hand blender (Philips) at full speed in 4 vol. of buffer A (0.05 M MOPS pH 6.5, 5 mM Na_2_EDTA, complete protease inhibitors tablets). The homogenate was strained through a filter using a cafetiere jug (Sainsbury's), and 4 layers of gauze tissue. It was centrifuged in a Ti45 rotor in an Optima^TM^ L-100 XP Ultracentrifuge (Beckman Coulter, Inc.) at 36,000 × *g* for 30 min, at 4 °C. Pellets were pooled, homogenised using the hand blender (Philips) in 4 vol. of buffer A, and centrifuged again as before to give crude membranes. Membranes were homogenised in 0.5 vol. of buffer B (0.05 M MOPS, pH 6.5), and stored in liquid nitrogen.

### 2.3 Isolation of HEK-293 membranes

Suspended cells were harvested at 2500 × *g* for 5 min and then broken by passage (2–3 times) through a 0.22 mm gauge needle in buffer B. Membranes were collected by centrifugation in the TLA55 rotor of the Optima^TM^ MAX Benchtop Ultracentrifuge (Beckman Coulter, Inc.) at 25,000 × *g* for 10 min at 4 °C.

### 2.4 Solubilisation of CHO and eel membranes

Membranes were solubilised at a detergent to protein ratio of 2 (w/w) for 1 h on ice in buffer B, if not stated otherwise. The solubilised membranes were directly used in the ligand-binding assays. Protein concentration was determined by absorption at 280 nm in 5% DDM.

### 2.5 Radioligand binding

Samples were incubated in buffer B with 0.5 nM tritiated saxitoxin (^3^H-STX), for 30 min at 0 °C. This time was sufficient to reach equilibrium as association and dissociation of ^3^H-STX/ STX (TTX) to VGSCs is fast [35]. All other steps were done at 4 °C. Toxins were used at concentrations 0.1 μM (PbTx-2), 0.5 μM (HuwTx-IV, HaTx-III), 1 μM (TTX, BTX, KTX, ProTx-II), 5 μM (ATX-II), and 100 μM (VTD). Nonspecific binding was determined in the presence of 2 μM TTX. Amounts of bound ^3^H-STX were determined by liquid scintillation counting in 3 ml OptiPhase Supermix (Perkin Elmer). Bound ^3^H-STX was determined using Tri-Carb 2910-TR liquid scintillation analyzer (Perkin Elmer) (30 min per sample). Data were plotted using the program GraphPad Prism (GraphPad Software, Inc.). Due to the availability of only limited quantities of membranes, other than from eel electroplax, some measurements could be done only with single points; however more than 10 single points per curve produced acceptable fitted curves using GraphPad Prism software.

#### 2.5.1 Assay for membrane-bound VGSCs (“membrane pellet assay”)

Membranes were incubated at 0 °C in buffer B at protein concentration 3 mg/ml in a total volume of 0.5 ml, and pelleted using a TLA55 rotor in a Optima™ MAX Benchtop Ultracentrifuge (Beckman Coulter, Inc.) at 45,000 × *g* for 30 min, at 4 °C. Specifically bound ^3^H-STX was determined in the pellet after subtraction of the nonspecific binding measured by displacement using unlabelled TTX.

#### 2.5.2 Assays on solubilised VGSC (“equilibrium ligand-binding assay” and “kinetic ligand-binding assay”)

Assays were performed by centrifugation chromatography on two different kinds of spin-column in the presence of 0.13% detergent at an initial detergent to protein ratio of 2 (w/w) unless stated otherwise. Bound ^3^H-STX was determined in the flow-through. Specific binding was measured by subtracting the total bound ^3^H-STX from the nonspecifically bound material measured in a parallel assay in the presence of 2 μM TTX. In the *“equilibrium ligand-binding assay”,* bound radioligand was measured by gel filtration with Quik-sep columns packed with 2 ml Sephadex G25F as in [16], modified so that the column was pre-equilibrated with the incubation buffer containing the (radio)ligand and using a sample volume of 170 μl (0.5–0.85 mg of eel membranes). In the “*kinetic ligand-binding assay*”, bound and free radioligand were separated by ion exchange chromatography with mini-spin columns packed with 0.25 ml CM Sepharose pre-equilibrated with buffer B containing the appropriate detergent. A 30 μl sample in the form of a single droplet (19 or 5 μg of eel membranes solubilised or non-solubilised, respectively, and 144–384 μg of solubilised CHO membranes) was loaded onto the lid of the column in a cold room, and centrifuged in FA-45-18-11 rotor (Eppendorf) at 21,000 × *g* for 1 min, at 4 °C. In the “*mini kinetic ligand-binding assay*”, bound and free radioligand were separated by gel filtration chromatography using Zeba Micro Spin Desalting Columns, pre-equilibrated with buffer B containing the appropriate detergent. In this case 15 μl sample in the form of a single droplet was loaded onto the lid of the column in a cold room, and centrifuged in FA-45-18-11 rotor (Eppendorf) at 21,000 × *g* for 1 min, at 4 °C. For the *thermostability assay,* non-solubilised or solubilised membranes were incubated for 30 min at the specified temperature in a total volume of 40 μl, and subsequently analysed by radioligand binding.

## 3 Results

### 3.1 Development of thermostability ligand binding assays for membrane-bound and detergent-solubilised VGSCs

**Fig. 1:**
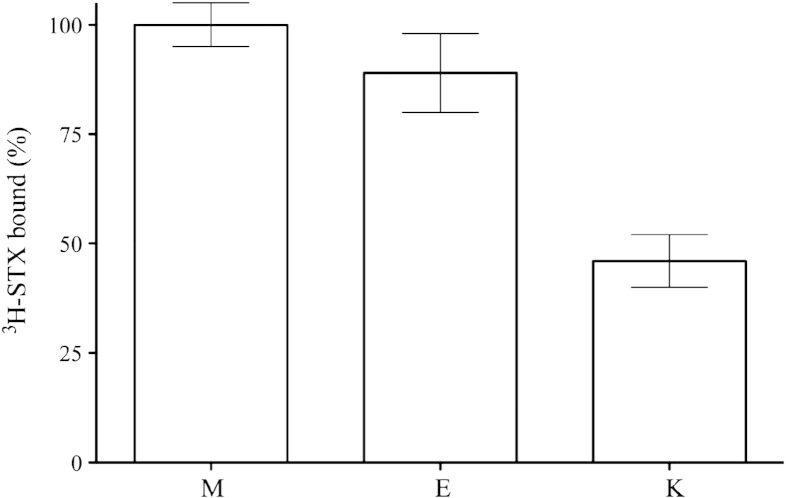
Comparison of the efficiency of three ligand-binding assays for VGSCs. Non- or detergent solubilised membranes were incubated with 0.5 nM ^3^H-STX for 30 min at 0 °C. The amount of ^3^H-STX binding to solubilised membranes was assessed by the equilibrium (E) and the mini kinetic (K) ligand-binding assays in comparison with the membrane pellet assay (M) using non-solubilised *E. electricus* membranes as a reference (100%). Presented data show an average values ± SEMs from a representative experiment done in duplicate. 100% corresponds to binding of 90410 dpm per mg membrane protein using 0.5 nM ^3^H-STX.

Three different assays were developed to quantify VGSCs, namely the membrane pellet assay, the equilibrium assay, and the kinetic assay. The first assay was used for membrane samples and the latter two assays for detergent-solubilised samples. Using the membrane pellet assay 40 pmol of VGSC was detected per mg of membranes. After detergent solubilisation either 35 or 20 pmol of VGSC was detected using the equilibrium or the kinetic assay, respectively. This corresponds to approximately 1% of the protein in membranes or in detergent extracts of electroplax membranes. Compared with the membrane pellet assay, we could recover 90% and 45% of radioligand binding in solubilised membranes measured by the equilibrium and kinetic ligand-binding assays, respectively (Fig. 1). The binding of ^3^H-STX could be eliminated by competition with an excess of unlabeled STX or tetrodotoxin (TTX) in the micromolar range (1–2 μM), demonstrating the signal-to-noise characteristics of the assay, which was 5:1, 2:1, and 10:1 for the membrane pellet, the equilibrium and the kinetic ligand-binding assays, respectively. The lower signal-to-noise ratio for the first two assays resulted from the higher background in the non-washed pellet and from pre-equilibration of gel filtration column with the radioligand, for the membrane pellet and the equilibrium ligand-binding assay, respectively.

### 3.2 Saxitoxin affinity to eel membrane-bound and detergent-solubilised VGSC

**Fig. 2:**
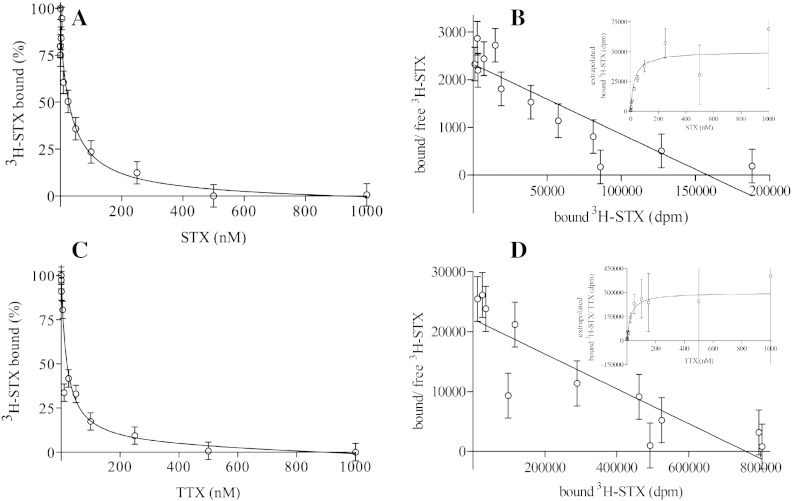
Binding affinity of STX and TTX to *E. electricus* VGSCs. Membranes were incubated with DDM:membrane protein ratio 2 (w/w) for 1 h, then displacement of ^3^H-STX by increasing concentrations of unlabeled STX (A) and TTX (C) in membrane-bound (A) and DDM-solubilised (C) VGSC was investigated. Binding of ^3^H-STX (saturation curve; B, D insets) was calculated by renormalizing the raw dpm measurements assuming reduced STX specific activity due to addition of unlabelled toxin using data A (B) and data C (D) with Scatchard plot (B, D) made by hand. Data were obtained with the membrane pellet (A, B) and the equilibrium ligand-binding assay (C, D). The experiment was done three times in singles. Presented data are from a representative experiment. Error bars of ± 6% (A) and 5% (C) were estimated from the average difference between points and a fitted curve calculated by GraphPad Prism. Error bars (5%) in B and D were propagated using the same factor as for the plotted points. In B and D, the different scale occurs because in B samples of 0.037 mg were used whereas in D samples of 0.69 mg were used.

Displacement of ^3^H-STX by unlabelled STX (Fig. 2A) and the saturation isotherm with ^3^H-STX (Fig. 2B) showed that STX binds with a high affinity to eel membrane-bound VGSC with K_D_ = 24 ± 2 nM, assuming that there is one binding site. Competition experiment with unlabelled tetrodotoxin (displacement of ^3^H-STX by unlabelled TTX, Fig. 2C; the saturation plot, Fig. 2D) showed complete competition, and indicated that in DDM-solubilised membranes TTX binds to VGSCs with K_D_ = 21 ± 2 nM, assuming that there is one binding site.

### 3.3 Stability of electric eel VGSC in the membrane and in detergents

**Fig. 3:**
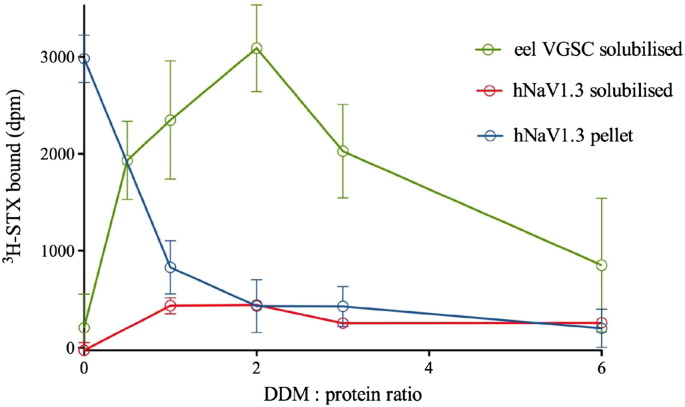
Detergent sensitivity of VGSCs at different detergent:membrane protein ratios. Sonicated membranes were incubated with various concentrations of DDM for 1 h, then the amount of ^3^H-STX binding to membranes solubilised with increasing ratios of DDM:protein from *E. electricus* (green circles) and CHO cells stably expressing hNaV1.3 VGSCs (red circles) and to the non-solubilised component of hNaV1.3 (blue circles). The decreased binding at high detergent:protein ratios shows how the integrity of the channels decreases at high detergent ratios. Data were obtained using the mini kinetic ligand-binding assay, and due to high cost of the experiment they are from one experiment done in duplicates (hNaV1.3), and from four experiments done in singles or duplicates (eel VGSC). The graphs shows mean value ± SEM, normalised for 0.146 mg of total membrane proteins for both, eel and hNaV1.3.

**Fig. 4:**
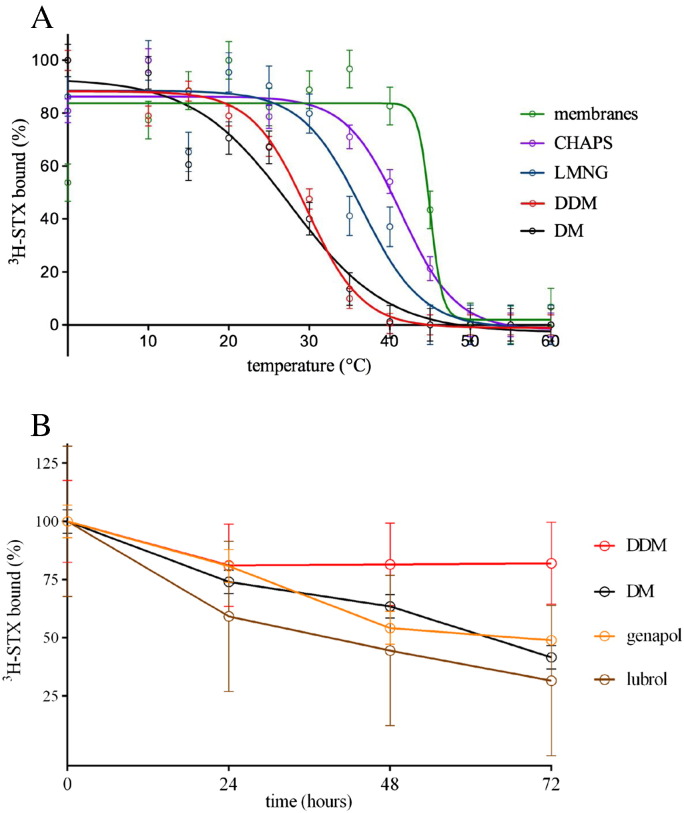
Stability of solubilised eel VGSCs in different detergents. (A) The stability was assessed by incubating aliquots of detergent-solubilised material at different temperatures for 30 min (apparent Tm in parentheses): black circles, DM (28 °C), red circles, DDM (30 °C); blue circles, LMNG (37 °C); purple circles, CHAPS (41 °C). The amount of detergent-solubilised eel VGSC was assessed by ^3^H-STX binding versus temperature. For comparison with membrane-bound channels, sonicated electroplax membranes were also incubated at different temperatures for 30 min; green circles (45 °C). Data were obtained using the kinetic ligand-binding assay. Each data point was determined in singles from a single experiment, but 12 points per curve enabled us to obtain good fits with values of *R*^2^ in the Prism software of 0.9251, 0.9462, 0.9844, 0.9786, and 0.9542 for membranes, LMNG, DDM, CHAPS, and DM, respectively. Error bars of ± 7.1%, 7.4%, 3.8%, 4.5%, and 6.1% were estimated from the average difference between points and a fitted curve calculated by GraphPad Prism for membranes, LMNG, DDM, CHAPS, and DM, respectively. (B) Detergents were added to electroplax membranes to give a final concentration of 1%, incubated for 1 h at 0 °C and the amount of detergent-solubilised VGSC was assessed by ^3^H-STX binding versus time at 4 °C after solubilisation at 0 °C: red circles, DDM; black circles, DM, orange circles, genapol; brown circles, lubrol. Data were obtained using the equilibrium ligand-binding assay. Each data point was determined in singles from either single or two independent experiments (0 h incubation time). The error bars for all data values were assumed to be the same as for the 0 h incubation time point, which was measured in duplicate.

Measurement of the binding of ^3^H-STX to solubilised VGSC from eel electroplax membranes in the presence of increasing amounts of the detergent dodecylmaltoside (DDM) is shown in Fig. 3. We found that the best solubilisation efficiency for eel VGSC was obtained at a detergent to protein ratio of around 2 (w/w). Higher amounts of detergent resulted in subsequent loss of overall ^3^H-STX binding, and lower amounts of detergent resulted in incomplete solubilisation (Fig. 3). In other detergents, the solubilisation behaviour of the VGSCs was similar but the stability after solubilisation depended strongly on the detergent used. As expected, VGSCs were most stable in their normal lipid environment within membranes in the absence of any detergent (apparent Tm in parentheses, 45 °C), and solubilised VGSCs had significantly higher Tm in LMNG or in CHAPS than in DDM (Fig. 4A). The order of temperature stability in detergents was (apparent Tm in parentheses) CHAPS (41 °C) > LMNG (37 °C) > DDM (30 °C) > DM (28 °C). The stability during a time series in lubrol or genapol was lower than the stability in DDM, and similar to the stability in DM (Fig. 4B). In DDM, the protein lost approximately 10% of its activity, and in DM, lubrol, or in genapol it lost approximately 50% of its activity during 3 days at 4 °C. Solubilised VGSCs were 1.2 or 1.4 times more stable in LMNG and CHAPS than in DDM at a fixed temperature (Fig. 4A), which demonstrates the improved stability in a range of new detergents that have been developed since the original studies [36].

### 3.4 Stability of mammalian (human, rat) VGSCs in detergents

**Fig. 5:**
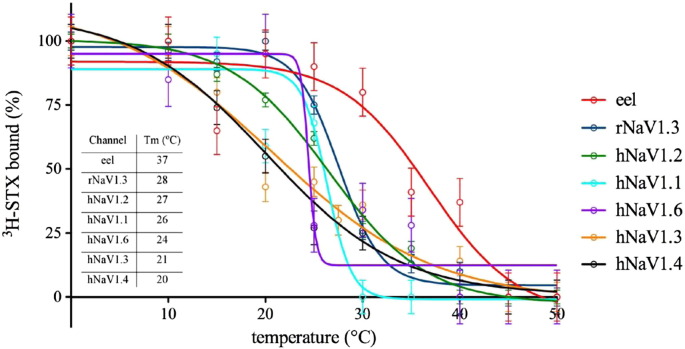
Thermal stability of mammalian VGSCs comparing to *E. electricus* VGSCs in detergent. Membranes were incubated with LMNG for 1 h, then the amount of ^3^H-STX binding to membranes solubilised from *E. electricus* and CHO cells stably expressing VGSCs was examined. The stability was assessed by incubating aliquots of detergent-solubilised channel at different temperatures for 30 min (apparent Tm in parentheses): dark blue circles, rNaV1.3; light blue circles, green circles, orange circles, black circles, and violet circles for hNaV1.1–1.4, and hNaV1.6, respectively. Comparative data for *E. electricus* VGSC are shown in red circles. Data were obtained using the mini kinetic ligand-binding assay. Due to high cost of CHO membranes expressing VGSCs, the data are from a single set of experiments. However, 10 points per curve enabled us to obtain good fits with Prism *R*^2^ of 0.9397, 0.9914, 0.9546, and 0.9581 for hNaV1.1–1.4 respectively, 0.9078 for hNaV1.6, 0.9909 for rNaV1.3, and 0.9124 for *E. electricus* VGSC. Error bars of ± 6.5%, 2.7%, 5.7%, and 6.6% were estimated from the average difference between points and a fitted curve (calculated by GraphPad Prism) for hNaV1.1–1.4 respectively, 10.5% for hNaV1.6, 3.6% for rNaV1.3, and 9.3% for *E. electricus*.

The best solubilisation efficiencies for the human VGSCs were also obtained at detergent to protein ratios between 1 and 2, with higher amounts of DDM resulting in subsequent loss of radioligand binding, showing how the integrity of the channels decreases at high detergent to protein ratios (Fig. 3). We chose human NaV1.3 for the detergent stability test within all recombinant human VGSCs available, as we found that it gives the highest signal measured by the kinetic radiochemical assay per 1 mg of membranes, so with those membranes we had a chance to test more experimental conditions. In VGSCs, similar to eel VGSC, lower amounts of detergent resulted in incomplete solubilisation of human VGSC from CHO membranes. Therefore a successful solubilisation of the channel has to be a compromise between the yield of the channel released from the membrane and possibilities to keep it in an active solubilised form. Different isoforms of mammalian VGSCs showed different stability, and the order of thermostability in LMNG was (apparent Tm in parentheses): rNaV1.3 (28 °C) > hNaV1.2 (27 °C) > hNaV1.1 (26 °C) > hNaV1.6 (25 °C) > hNaV1.3 (23 °C) > hNaV1.4 (22 °C) (Fig. 5). Compared with the eel toxin-binding site (Tm, 37 °C in LMNG), human VGSCs are less stable at the same detergent to protein ratio, and are less stable with increasing temperature (Fig. 5). Although hNaV1.6 is not identified as the most stable VGSCs within all mammalian VGSCs investigated, it has, as the only one, a steep Tm curve.

### 3.5 Stabilising effect of different toxins

**Fig. 6:**
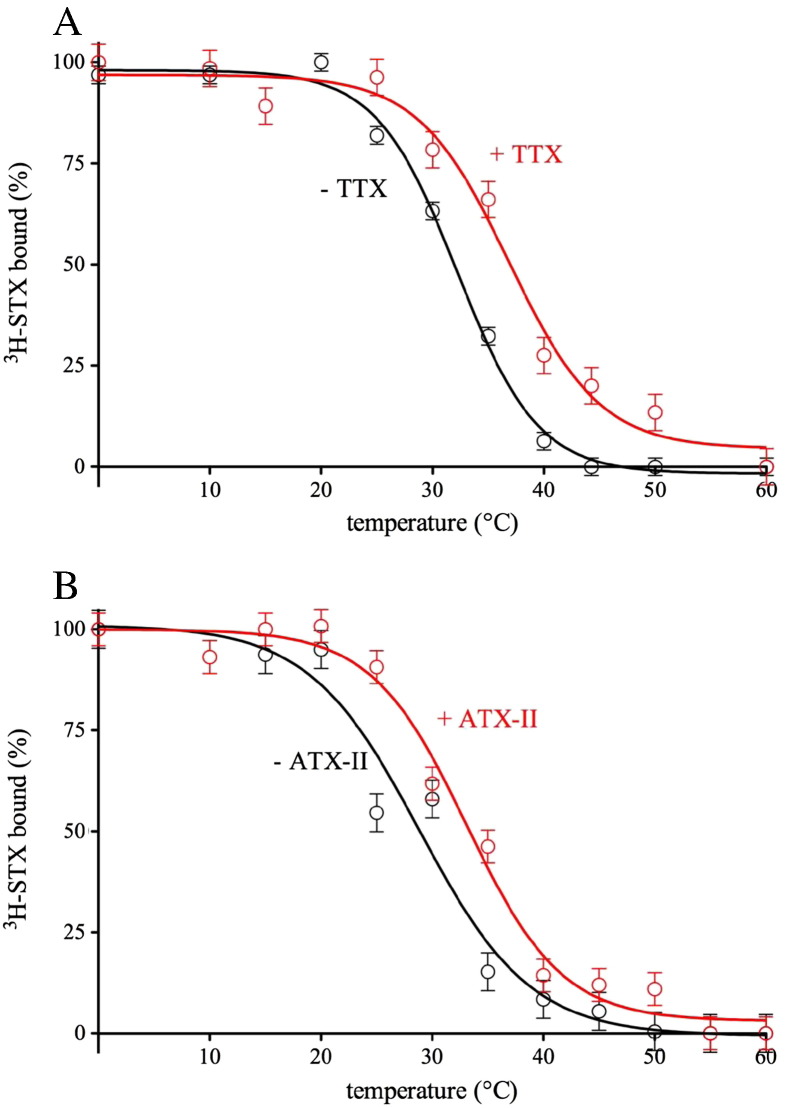
Stabilizing effect of TTX and ATX-II on *E. electricus* VGSCs in DDM-solubilised membranes. The stability was assessed by incubating aliquots of detergent-solubilised channel at different temperatures for 30 min (apparent Tm in parentheses) in the presence (red squares) or absence (black circles) of: (A) TTX or (B) ATX-II. The experiment was done twice in singles by the equilibrium and by the mini kinetic ligand-binding assay. Presented data are from a representative experiment obtained by the equilibrium ligand-binding assay with Prism *R*^2^ of 0.9842 and 0.9879 for (A) and (B), respectively. Error bars of ± 4% were estimated from the average difference between points and a fitted curve (calculated by GraphPad Prism) for both, (A, + TTX) and (B, + ATX-II).

We found that among several different toxins tested (ATX-II, BTX, HaTX, HuwTX, KTX, PbTx-2, ProTx-II, TTX, VTD), TTX (Fig. 6A) and ATX-II (Fig. 6B) had a stabilising effect on detergent-solubilised VGSCs at concentrations of 0.5 and 5 μM, respectively. Both TTX and ATX-II ligands improved thermostability of eel VGSC (measured by apparent Tm) by 4–5 °C (Fig. 6A, B). For other toxins tested neither significant stabilizing nor destabilizing effect on ^3^H-STX binding was observed (data not shown).

## 4 Discussion

The availability of pure, stable and well-behaved protein is the key factor for successful structural studies for all membrane protein structural analyses. *In vivo*, human VGSCs require only low levels of natural expression to fulfil their function of triggering an action potential, but neither a robust recombinant expression system nor a purification protocol to produce suitable samples of human VGSCs for structural studies has been reported so far. There are a number of mammalian, and at least nine human VGSCs for which structural data are desired. Therefore, with the ultimate goal of high-resolution structure of eukaryotic VGSCs, we have investigated the stability of human and rat VGSCs in a solubilised state and compared them with electric eel VGSC. We hoped to identify the most suitable human VGSC, to use as a platform to establish biochemical or stabilisation protocols, such as the method of conformational stabilisation [17] used so successfully for G protein-coupled receptors (GPCRs) [16]. An assay to follow the progress of expression and purification of functional protein is an absolute key to study VGSCs. The present work was therefore designed to build on earlier efforts to use radiolabeled toxin binding to characterize the expression levels of functionally competent VGSCs, with an eventual goal of obtaining high level expression of the most stable channels. In [36] VGSCs were assayed by ^3^H-TTX binding in detergent extracts following a gel filtration procedure. However, at present radiolabeled TTX of high specific activity is not commercially available, and our attempts to repeat published procedures [37] were unsuccessful. Therefore we established three different radioligand binding assays using commercially available ^3^H-STX, namely the membrane pellet, the equilibrium and the kinetic ligand-binding assays. These three assays may be used to assess receptor abundance in membrane-bound or detergent-solubilised samples in which ligand dissociation rates and receptor abundance are unknown. Despite the fact that STX binds with high affinity and is competitively displaced by TTX, the rates of dissociation are known to be much faster than for TTX [35,36,38]. Therefore an equilibrium assay was a necessary aspect of the binding measurements. The first two assays, the membrane pellet and the equilibrium ligand-binding assay are both equilibrium methods usable for assessment of membrane-bound and detergent-solubilised VGSCs, respectively, which are not influenced by the TTX or STX on- or off-rates. A relatively high nonspecific background in the assay does not affect the assessment of ligand binding to receptors in eel with a tissue that is abundant in a single isoform of Na^+^ channel. However the expression levels of recombinant VGSCs are still very low, so equilibrium ligand-binding assays are not useful to follow recombinant expression, e.g. in HEK-293 or CHO cells. That is why it was necessary to use a third type of assay, sensitive to STX kinetics in VGSCs, which we called the kinetic ligand-binding assay. In the kinetic assay, in which bound and free ^3^H-STX are separated by ion exchange chromatography (free ^3^H-STX binds to the cation-exchange resin, and bound ^3^H-STX passes through the column together with the receptor), the aim is to separate the bound from free radioligand in a few seconds before there is time for a significant amount of dissociation to occur. The advantage of the assay is that signal-to-noise ratio is relatively high, so the assay is able to detect even small amounts of detergent-solubilised receptors. From Fig. 1, it can be seen that the separation was indeed fast enough to measure binding, but the amount of bound ligand was slightly less than expected, so a small amount of ^3^H-STX appears to have dissociated during the spin. An approximate 50% loss of 3H-STX binding upon passing through the spin column in the kinetic assay is consistent with STX off-rates, which have been measured previously in [35] by direct binding experiments on solubilised garfish nerve membranes.

Binding of ^3^H-TTX to detergent extracts of electroplax membranes was studied previously by Agnew et al. [36], where authors reported Kd values of 1 and 10 nM for TTX and STX, respectively for a single class of binding sites. The observed higher affinity of TTX than STX to eel VGSC fits with the reported data [36], however the reported affinity is higher than we found for non- and detergent solubilised membranes (24 and 21 nM, for STX and TTX respectively). The observed lower affinity of STX might be explained by the influence of the lipid environment on the binding properties of VGSCs, as we investigated saxitoxin affinity for VGSCs within membranes (non-solubilised state). The affinity can be influenced by the lipids as well as by the detergent environment. In our study we used DDM to investigate the affinity of tetrodotoxin, whereas Agnew et al. [36] studied the affinity of TTX and STX in the presence of lubrol. Different solubilisation conditions (detergent concentration, buffer system), and finally different physical properties of a ligand-binding assays used in both studies may explain the slightly lower affinity of the VGSCs observed here. The stabilising effect of TTX on toxin-binding site in eel electroplax was reported previously [36,39,40]. We also found that the peptide neurotoxin isolated from the sea anemone (ATX-II) can stabilise eel VGSC, whereas none of the other toxins tested produced either measurable stabilizing or destabilizing effects on ^3^H-STX binding. Thus, TTX and ATX-II would be valuable additives during purification and for any subsequent structure analysis via crystallization or electron cryomicroscopy.

The ratio of detergent to protein is a critical factor that affects solubilisation and stability of membrane proteins. On one hand, the protein must be released from a membrane, but on the other hand its structure must be intact, so that it remains functionally active. In previous attempts at structural work [12,39], no assays to show retention of function in the purified material of any kind were carried out. We found that human VGSCs are less stable after solubilisation than eel VGSCs. DDM-solubilised eel VGSCs were previously reported [39], as well as the solubilisation using lubrol [12], SDS [40], CHAPS [41], genapol [13] or NP-40 [42]. To choose the most promising detergent to solubilise the protein, we analyzed time stability and thermostability of eel VGSC in different detergents. The specific activity of 20–35 pmol/ mg after solubilisation is similar to that observed previously [e.g. 36]. However, the use of LMNG, which was not reported so far to solubilise VGSCs, may be more promising for solubilisation of receptors from electroplax, either alone or in combination with, for example CHAPS. However, as reported earlier, the detergent-solubilised membrane protein is much less stable [36,41,44] than it is in the membrane environment prior to solubilisation, and binding activity is rapidly lost at 37 °C [44] together with the initial loss of helical structure starting at temperature above 40 °C [39]. Although Charalambous et. al. [39] reported successful refolding the thermally denatured eel VGSC analysed by CD spectroscopy, our data obtained by the radiochemical binding assay clearly show that the channel even surrounded by the native lipid environment, loses its ligand binding activity at temperatures above 40 °C. Eel VGSCs are more stable in detergent than those mammalian VGSCs that we have examined, so eel would be our first choice for structural studies of VGSCs to provide the foundations for future insight into human VGSC structural studies.

As long as mutagenesis studies are not done, it is not clear why eel VGSC is more stable than all mammalian VGSCs examined (especially human NaV1.4 which has the closest homology), and again, why rat NaV1.3 is more stable than all human VGSCs examined. We can only speculate on a basis of simple bioinformatics tools like Clustal or Ronn, that different stability of VGSCs may be related to different size of their extra- and intracellular loops responsible for disorder regions. For example, within all the VGSCs analysed, human NaV1.4 has the longest loop between TM5DI and TM6DI and the eel VGSC has the shortest one. However, this does not really mean that the shorter loops will always give more stable VGSC, because human NaV1.4 contains the shortest TM6DI/ TM1DII loop within all VGSCs investigated and comes out as one of the least stable. On the other hand, rat NaV1.3 has a high sequence identity (94.8%) with human NaV1.3, and the main difference is that rat NaV1.3 has a shorter intracellular loop TM6DI/TM1DII than human. A steep melting curve may signal a higher potential for protein stability improvement. It is possible that the C-terminus affords VGSC stability. For example, human NaV1.6 has a steep melting curve and is the only VGSC investigated which has a unique hNaV1.6 C-terminus deletion.

At present, it appears that the best material for structural analysis is eel or rNaV1.3 VGSC, though the possible approaches to structure may be limited to cryoEM in the short term. Human VGSCs will require further stabilisation either by systematic mutagenesis [17] or the use of novel solubilisation methods such as new detergents [45] or amphipols [43] before structural analysis can be tackled by a wider range of methods as crystallisation and X-ray crystallography.

## Transparency document

Transparency document
